# Identification of *BRCA2 Cis* Double Heterozygous Breast Cancer Cases Using Whole Exome Sequencing: Phenotypic Expression and Impact on Personalized Oncology

**DOI:** 10.3389/fgene.2021.674990

**Published:** 2021-08-12

**Authors:** Yosr Hamdi, Maroua Boujemaa, Najah Mighri, Nesrine Mejri, Olfa Jaidane, Sonia Ben Nasr, Hanen Bouaziz, Jamel Ben Hassouna, Aref Zribi, Yossra Berrazaga, Haifa Rachdi, Nouha Daoud, Houda El Benna, Soumaya Labidi, Abderrazek Haddaoui, Khaled Rahal, Farouk Benna, Hamouda Boussen, Sonia Abdelhak, Samir Boubaker

**Affiliations:** ^1^Laboratory of Biomedical Genomics and Oncogenetics, LR20IPT05, Institut Pasteur de Tunis, University of Tunis El Manar, Tunis, Tunisia; ^2^Laboratory of Human and Experimental Pathology, Institut Pasteur de Tunis, Tunis, Tunisia; ^3^Medical Oncology Department, Abderrahmen Mami Hospital, Ariana, Tunisia; ^4^Surgical Oncology Department, Salah Azaiez Institute of Cancer, Tunis, Tunisia; ^5^Department of Medical Oncology, Military Hospital, Tunis, Tunisia; ^6^Department of Radiation Oncology, University of Tunis, Tunis, Tunisia

**Keywords:** breast cancer susceptibility, whole exome sequencing, *Cis* double heterozygosity, BRCA2 mutations, early age of onset, phenotype genotype correlation

## Abstract

*BRCA1* and *BRCA2* are the most commonly mutated breast cancer susceptibility genes that convey a high risk of breast and ovarian cancer. Most *BRCA1* or *BRCA2* mutation carriers have inherited a single heterozygous mutation. In recent years, very rare cases with biallelic or trans double heterozygous mutations on *BRCA1* and or *BRCA2* have been identified and seem to be associated with distinctive phenotypes. Given that this genotype-phenotype correlation in cancer predisposing hereditary conditions is of relevance for oncological prevention and genetic testing, it is important to investigate these rare *BRCA* genotypes for better clinical management of *BRCA* mutation carriers. Here we present the first report on *Cis* double heterozygosity (*Cis* DH) on *BRCA2* gene identified using Whole exome sequencing (WES) in a Tunisian family with two *BRCA2* mutations namely: c.632-1G>A and c.1310_1313DelAAGA that are both reported as pathogenic in ClinVar database. Subsequent analysis in 300 high-risk Tunisian breast cancer families detected this *Cis* double heterozygous genotype in 8 additional individuals belonging to 5 families from the same geographic origin suggesting a founder effect. Moreover, the observed *Cis* DH seems to be associated with an early age of onset (mean age = 35.33 years) and severe phenotype of the disease with high breast cancer grade and multiple cancer cases in the family. The identification of unusual *BRCA* genotypes in this Tunisian cohort highlights the importance of performing genetic studies in under-investigated populations. This will also potentially help avoiding erroneous classifications of genetic variants in African population and therefore avoiding clinical misdiagnosis of *BRCA* related cancers. Our findings will also have an impact on the genetic testing and the clinical management of North African breast cancer patients as well as patients from different other ethnic groups in regard to several emerging target therapies such as PARP inhibitors.

## Introduction

Breast cancer is the most common malignancy in women worldwide (Rojas and Stuckey, 2016). Incidence and mortality rates of breast cancer differ between populations (Bray et al., 2018). Moreover, remarkable diversity in epidemiological and genetic characteristics of this disease have been observed (Servick, 2014). Since the mid-1990, extensive efforts have been dedicated to the analysis of *BRCA1* and *BRCA2*, the two most commonly mutated breast cancer genes. So far, about 1,800 mutations have been identified on *BRCA1* and almost 2,000 mutations on *BRCA2* (Szabo et al., 2000). These mutations explain around 20–30% of hereditary breast cancer cases and seem to be associated with different other cancers such as ovarian, prostate, colorectal, pancreatic, and melanoma (Mehrgou and Akouchekian, 2016). Indeed, the identification of a *BRCA* mutation can lead to risk or mortality reduction if optimal surveillance, risk-reducing mastectomy, and risk-reducing salpingo-oophorectomy are applied (Finch et al., 2006; Domchek et al., 2010). In addition, cancer treatment of *BRCA* mutation carriers has advanced with the development of PARP inhibitors, which take advantage of the loss of *BRCA1/2* function in tumors (Farmer et al., 2005).

The vast majority of *BRCA1* and *BRCA2* mutation carriers are single heterozygous (SH) with only one mono-allelic deleterious mutation on one of these two genes. Excluding individuals of Ashkenazi descent, it is uncommon to identify carriers of two deleterious mutations either within the same gene [biallelic or double heterozygous (DH)] or in both genes [trans heterozygous (TH)]. Indeed, biallelic mutations in *BRCA2* results in childhood Fanconi anemia (FANCD1) (Alter et al., 2007), which is an autosomal recessive disease resulting in developmental abnormalities, bone marrow failure, and early-onset leukemia or solid tumors. The biallelic *BRCA2* genotype has also been observed in a 31-year-old female diagnosed with colorectal cancer (Degrolard-Courcet et al., 2014). Biallelic mutations in *BRCA1* are much rarer because carrying the same *BRCA1* pathogenic mutation on both alleles is widely known to be embryonic lethal (Evers and Jonkers, 2006). However, Domchek et al. (2013) reported a case with biallelic *BRCA1* mutations diagnosed with an ovarian cancer at age 28 with short stature, microcephaly, developmental delay, and significant toxicity from chemotherapy. Another reported case of biallelic *BRCA1* mutations was in a woman with a Fanconi anemia disorder and breast cancer at age 23 (Sawyer et al., 2015). TH mutations in both *BRCA1* and *BRCA2* genes have been also reported in almost 68 cases and seem to be associated with an early age of onset and a severe disease compared to SH *BRCA* mutation carriers (Heidemann et al., 2012; Rebbeck et al., 2016). However, *Cis* double heterozygosity, have been reported in only two studies with controversial results (Colombo et al., 2009; Pensabene et al., 2009). Here we define the *Cis* DH to be the inheritance of two deleterious mutations on the same allele of a gene. To date, the phenotypic consequences of this unusual *BRCA* genotype are poorly understood.

In fact, trans biallelic *BRCA* deleterious mutations, either in one gene or one allele per gene, might be expected in families with both parental lineages affected by breast and/or ovarian cancer. However, *Cis* DH most likely increases cancer occurrence in one parental bloodline, albeit, in consanguineous and endogamous populations such as the Tunisian population, *Cis* DH may increase cancer incidence in both maternal and paternal sides. Moreover, intuitively, one can anticipate that *Cis* DH is not associated with additional phenotypic consequences over and above that associated with single heterozygosity since a single mutation is sufficient to completely abolish the synthesis of a functional gene product. Contrariwise, it has been recently shown that breast and ovarian cancer risks differ depending on the position and the type of *BRCA1* and *BRCA2* mutations (Rebbeck et al., 2015) meaning challenges regarding their structure and function. Thus, carrying two different mutations on the same allele may be associated with a distinctive phenotype since each mutation is located in a different domain of the BRCA protein and consequently could disturb the interaction of BRCA with several other proteins. Therefore, these altered protein-protein interactions may influence the resulting phenotype.

In the current study, we aimed to present a first report on a *BRCA2 Cis* double heterozygosity in 5 Tunisian families thanks to the use of whole exome sequencing. The founder and the functional effect of the two mutations have been assessed as well as the prevalence and the phenotypic spectrum of this unusual *BRCA2* genotype.

## Materials and Methods

### Patients

Tunisian families from different geographic origins were selected based on the following selection criteria; (1) having at least 3 breast cancer cases in first or second degree relatives at any age, (2) 2 breast cancer cases with one of them diagnosed with BC before age 40, (3) isolated breast cancer cases diagnosed before age 36, (4) one case with triple negative breast cancer (TNBC) at an age ≤ 40 years, (5) one case and one ovarian case diagnosed at first or second degree relatives at any age, (6) at least 2 cases with breast or ovarian cancer (at any age) and at least one case with pancreas cancer or prostate cancer at first or second degree relatives.

For each participant, total genomic DNA was extracted and used as a template for exome sequencing using DNeasy blood DNA extraction Kit (Qiagen) according to the manufacturer's instructions. DNA purity and concentration were measured using a NanoDrop™ spectrophotometer.

When possible, genomic DNA samples from other affected or unaffected family members were obtained for further validation. Written informed consent was obtained from all participants. The study was conducted according to the Declaration of Helsinki Principles. Ethical approval was obtained from the biomedical ethics committee of Institut Pasteur de Tunis (2017/16/E/Hôpital A-M).

### Data Analysis

**Figure 1:**
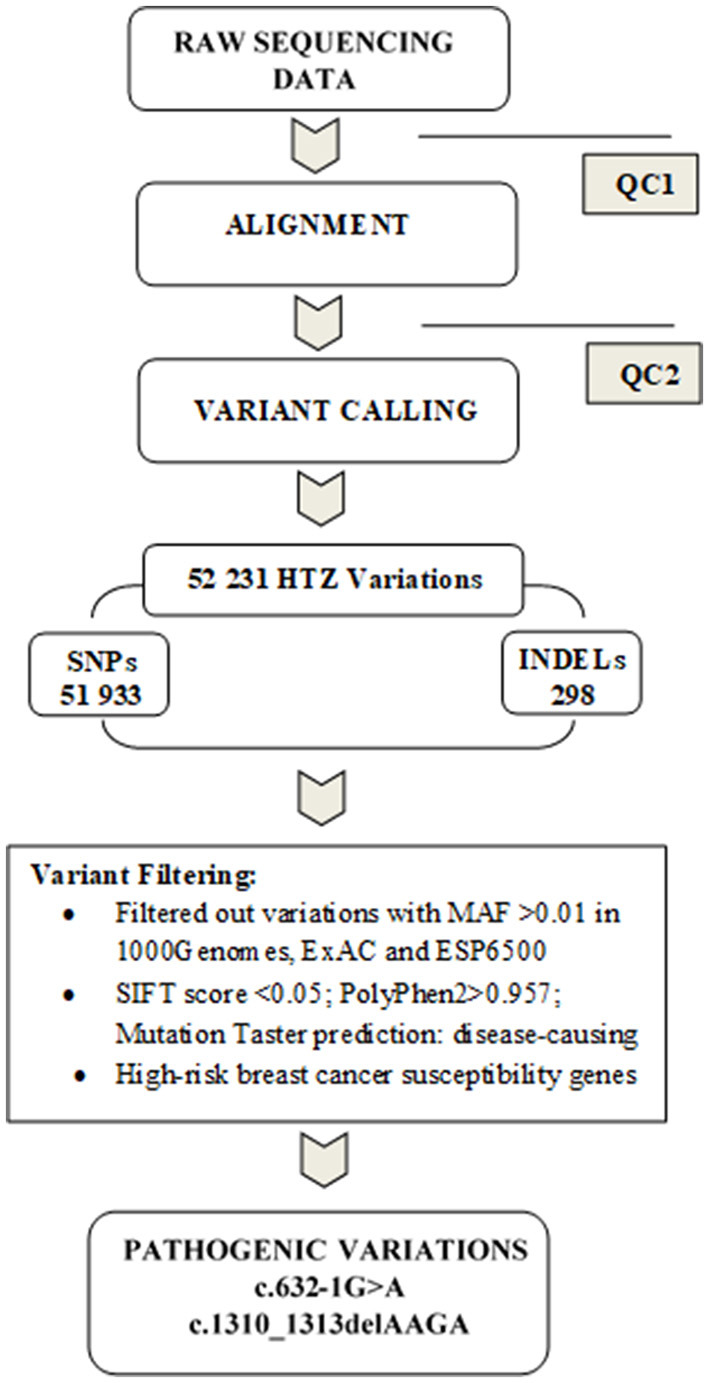
Summary of the data analysis pipeline followed in the present study. Before proceeding to the alignment phase, quality control (QC1) was performed on raw sequencing data in order to ensure that the data are optimal for downstream analyses. A second quality control (QC2) was done on alignment data to reduce the alignment artifacts that could influence the quality of downstream variant calling. Subsequently, variants were filtered by keeping only heterozygous variations and discarding those with MAF >1% in either Exac, 1,000 genomes or ESP6500. We further removed benign or tolerated variations and focused on genes related to breast cancer susceptibility. Two pathogenic mutations were identified c.632-1G>A and c.1310_1313delAAGA in *BRCA2* gene.

Data analysis pipeline is summarized in Figure 1. Exome sequences were mapped to their location in the most recent build of the human genome (hg19/b37) using the Burrows-Wheeler Aligner (BWA) software package version 0.7.5 (Li and Durbin, 2009). The subsequent SAM files were converted to BAM files using Samtools version 1.6 (Li et al., 2009). Duplicate reads were removed using Picard version 2.6 (http://broadinstitute.github.io/picard/). GATK version 4.1.2 (Van der Auwera and O'Connor, 2020) was then used to recalibrate the base quality scores, as well as, for SNP and short INDEL calling. Annotation and prioritization of potential disease-causing variants were performed using VarAFT (Variant Annotation and Filtering Tool) version 2.13 (Desvignes et al., 2018). To annotate variants, VarAFT uses ANNOVAR, a command line tool. INDELs and SNPs annotated were filtered according to several criteria: considering breast cancer as autosomal dominant disease, variants that were found in a homozygous state were removed. In addition, variants identified as intronic, intergenic, and synonymous were discarded. Then, assuming that causal variants are rare, we removed all variants with an allele frequency more than 1% either in ExAC version 0.3 (Lek et al., 2016), 1,000 genomes 2015-08 (Genomes Project et al., 2015) or ESP6500 (http://evs.gs.washington.edu/EVS/). We also removed all variants with no functional effect (annotated as “Benign” or “Tolerated” by the different tools used to predict the functional effect of these genetic variants). Finally, we used the literature to filter the remaining set of variants by gene function and only variants located on genes involved in cancer etiology were kept.

### Sanger Sequencing

Sanger sequencing technique was first used to validate the identified mutations resulting from the whole exome sequencing then to test the identified *BRCA2* variations in other family members or in other patients from our cohort. PCR reactions were performed on genomic DNA following standard protocols, followed by Sanger sequencing using an automated sequencer (ABI 3500; Applied Biosystems, Foster City, CA) and a cycle sequencing reaction kit (Big Dye Terminator kit, Applied Biosystems). Data were analyzed using BioEdit Sequence Alignment Editor Version 7.2.5. Sequencing was conducted using the following primers for exon 8 (8F: 5′-GCCATATCTTACCACCTTGT-3′ and 8R: 5′-CAGGTTTAGAGACTTTCTC-3′) and exon 10 respectively (10BF: 5′-CAGAAGCCCTTTGAGAGTGG-3′ and 10BR: 5′-GGTACCTGAATCAGCATTTGC-3′).

### Splicing Predictions

The c.632-1 G>A mutation is located at the splicing donor site of exon 8 of *BRCA2*.The functional effect of this mutation on splicing was evaluated using different splice-site *in silico* prediction tools: Human Splice-Finder (Desmet et al., 2009) (http://www.umd.be/HSF/) which is an online bioinformatics tool designed to predict the effects of mutations on splicing signals, Mutation Taster (Schwarz et al., 2010) (http://www.mutationtaster.org/) that evaluates disease-causing potential of sequence alterations including coding and non-coding variants and Alamut (https://www.interactive-biosoftware.com/fr/alamut-visual/). This latter integrates information from different public databases and several pathogenicity predictions tools to annotate variations. Human Splice-Finder and Mutation taster were used online while Alamut software was downloaded and used locally in order to predict the functional impact of the splice site variant.

### ClinVar Database

ClinVar (Landrum et al., 2020) is a public database of reports on the relationships between human variations and phenotypes, that uses supporting evidence to classify genetic variants based on their pathogenicity. As a freely accessible public database, we used ClinVar to assess the pathogenicity of the two mutations identified on exons 8 and 10 of the BRCA2 gene.

### RNA Extraction and RT-PCR Product Analysis

In order to assess the impact of the c.632-1 G>A mutation at the RNA level, total RNA has been isolated from peripheral blood using the Trizol method (Invitrogen™).

The cDNA was synthesized using random primers and SuperScript™ II Reverse Transcriptase (Invitrogen), according to the manufacturer's protocol.

In order to amplify the cDNA sample from two patients carrying the c.632-1G>A mutation and a control who is a breast cancer case that do not carry the mutation, we used four combinations of forward and reverse primer pairs flanking *BRCA2* exons 5–10 to amplify overlapping regions of the mRNA and to cover the entire open reading frame.

In order to evaluate the quality of the cDNA amplification, a PCR for a housekeeping gene *GAPDH* was conducted using the following primers (5′-GAGTCAACGGATTTGGTCGT-3′) and (5′-TTGATTTTGGAGGGATCTCG-3′).

RT-PCR products were separated on agarose gel and visualized by SYBR® Safe staining then purified using Exo-SAP and characterized by direct sequencing.

### Microsatellites Genotyping and Haplotype Analysis

Haplotype analysis was carried out on 60 individuals: 11 mutation carriers, 4 relatives, 8 breast cancer non-carriers, 24 breast cancer cases with unknown *BRCA* status and 13 healthy Tunisian women from the general population.

Four *BRCA2* extragenic polymorphic microsatellite markers, listed in order from centromere to telomere (D13S267, D13S171, D13S260, and D13S1246) and spanning a 6.43 Mb region around the *BRCA2* gene were studied. Characteristics of the studied region and analyzed markers are presented in Supplementary Figure 1.

PCR primer sequences were obtained from the Probe NCBI database. Genotyping technique is based on a fluorescent-labeled M13(-21) universal primer. The PCR mix contained 20 μmol/L of each forward and FAM-M13 primer and 2 μmol/L of the reverse primer in a final 25 μL reaction. PCR amplification conditions are as follows: 94°C (5 min), then 35 cycles at 94°C (45 s)/annealing temperature (30 s)/72°C (1 min 30 s), and a final extension at 72°C for 10 min.

One micro liter of the PCR product is added to 12 μl of formamide and 0.5 μl of GeneScan™ 500 ROX™ Size Standard and PCR product size was evaluated by capillary electrophoresis on ABI prism 3500 DNA Genetic Analyzer (Applied Biosystems, Foster City, CA). Data was analyzed using the GeneMapper V.5.0 software.

Haplotypes were reconstructed by PHASE v.2.1 software (Stephens et al., 2001), that used Bayesian methods to predict the haplotype distribution.

## Results

A 36 years old patient (BC17) consulted for a mass in the right breast. Clinical and radiological explorations concluded to a bilateral breast cancer. She had a bilateral mastectomy with right lymph node dissection. Histopathological examination concluded for the right breast to be an infiltrating ductal carcinoma, SBRIII, positive hormone receptors with 6 metastatic lymph nodes. In the left breast, there was an intraductal carcinoma. Adjuvant treatment was indicated and consisted in right loco-regional radiotherapy, chemotherapy, chemical ovarian function suppression and hormonal therapy by Tamoxifen. Eleven years later, she developed a squamous cell carcinoma of the esophagus. A radio-induced cancer was evoked and squamous cell carcinoma was confirmed. Total oesophagectomy by stripping was performed. No adjuvant treatment was indicated. One year later a recurrence of the cervical anastomosis was diagnosed and caused a severe dyspnea. Chemotherapy was rejected due to bad general conditions and the patient died of this recurrence. In addition to the severe phenotype of this patient, 9 family members have been diagnosed with breast cancer, 5 of them were diagnosed ≤ 45 years old. Therefore, a DNA sample of this patient was analyzed using whole exome sequencing. Results including number of reads, sample coverage, number of the different types of mutations and sequencing depth of the whole exome sequenced patient have been summarized in Table 1. This analysis unexpectedly revealed two *BRCA2* mutations corresponding to the frameshift c.1310_1313DelAAGA within exon 10 and a splice site mutation c.632-1G>A in exon 8.

**Table 1:** Exome sequencing data.

**Data**	**BC17**
Total reads	46,751,331
% Reads mapped to human genome	99.63
Total coverage	2,827,432,387
Mean read depth of target regions (X)	54.86
% Coverage of target regions (more than 10X)	91.1
% Coverage of target regions (more than 20X)	82.9
Number of SNPs (Heterozygous)	52,231
Number of coding SNPs	14,192
Number of splicing SNPs	383
Number of synonymous SNP	7,204
Number of non-synonymous SNPs	6,372
Number of non-frameshift INDELs	205
Number of non-frameshift substitutions	0
Number of frameshift INDELs	93
Number of stop gain SNPs	57
Number of stop loss SNPs	6

Interestingly, both mutations have been reported as “pathogenic” in the ClinVar database.

**Figure 2:**
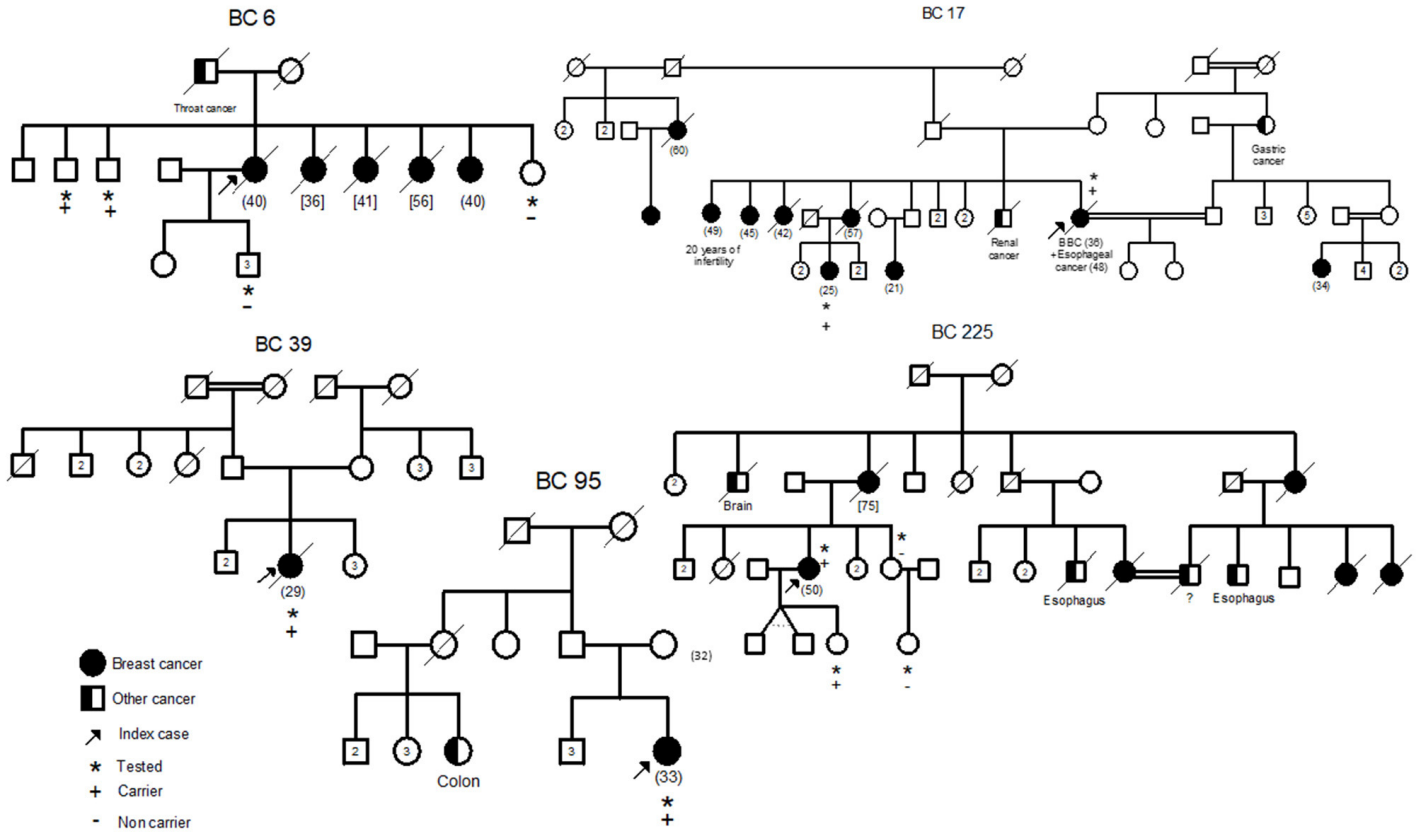
Familial pedigrees of the 5 *Cis* double heterozygous families. Available age at diagnosis of breast cancer cases is indicated between brackets. For some cases, only age at death was available and is indicated in square brackets. BBC, Bilateral Breast Cancer.

In addition, the two mutations have been confirmed by Sanger sequencing of the original DNA and of a second DNA sample extracted from a second blood sample of the same patient. These mutations were also confirmed in a relative of the patient diagnosed with pregnancy associated breast cancer at 25 years old (BC17-2). Subsequently, we searched for the 2 mutations in a cohort of 300 high risk Tunisian breast cancer families. Interestingly, 7 additional individuals belonging to 4 Tunisian families were identified as carriers of both mutations (BC6, BC39, BC95, and BC225). Pedigrees of these families are shown in Figure 2. Due to the fact that a subset of Fanconi Anemia patients carry biallelic *BRCA2* mutations, it was crucial to establish whether the two mutations are found *in Cis* or *in Trans*. Beyond the index case in the BC6 family, her son, her sister and two brothers have been tested for these mutations. The son and the sister were negative for both mutations and the two brothers have been identified as carriers of the two *BRCA2* mutations. This allowed us to conclude that both mutations are found on the same allele. Furthermore, 6 individuals belonging to 5 additional families have been identified as carriers of the founder c.1310_1313DelAAGA mutation only (PEC9, PEC35, BC245, BC354-1, BC354-2, DEP13-1) including 2 unaffected carriers.

The 9 carriers of this *Cis* DH *BRCA2* genotypes include 6 affected cases and 3 unaffected carriers.

Epidemiological and clinico-pathological data of these *Cis* DH families are reported in Table 2. The clinical phenotype correlates with the typical *BRCA2* tumor characteristics with positive estrogen and progesterone receptors and negative Her2 status. Moreover, T2 tumor size and a histological Grade III have been observed in 3 out of the 6 patients suggesting a severe phenotype for *Cis* DH *BRCA2* mutation carriers. In addition, the *Cis* DH *BRCA2* genotype seems to be associated with a significant early age of onset (mean age = 35.33 years) when compared to 48.1 in SH, 47.5 in *BRCA2* mutation carriers and 44.6 years in compound heterozygous. Furthermore, multiple breast cancer cases have been recorded in the related individuals of these *Cis* DH cases. No ovarian cancer was reported, which may not be surprising given the fact that ovarian cancer penetrance is significantly lower in *BRCA2* mutation carriers compared to *BRCA1*. Although prostate cancer is part of the *BRCA2* cancer susceptibility spectrum, no prostate cancer was reported as well. However, a brother of BC6 that has been tested positive for both mutations seems to have urological problems that may orient to a prostate pathology. One colon cancer was observed in the cousin of BC95, one brain cancer in the uncle of another case (BC225) and a renal cancer as well as a gastric cancer were diagnosed in a brother and an aunt of BC17, respectively. Even if they are not clearly *BRCA2* related carcinomas, esophageal and throat cancers were detected in 4 individuals from 3 different families (BC6, BC17, and BC225) suggesting an upper aero digestive cancer predisposition.

**Table 2:** Epidemiological and clinical data of *Cis* double heterozygous families.

**Patient ID**	**Pathology**	**Age at diagnoses**	**Family History**	**Histology**	**Histological grade**	**TNM**	**Lymph node status**	**Tumor size (mm)**	**ER**	**PR**	**HER2**	**Ki67**	**Treatment**	**Outcome**
BC6-1	BC (RB)	40	4 BC 1 ThC	IDC	NA	NA	NA	NA	NA	NA	NA	NA	Mastectomy, chemotherapy, and radiotherapy	Died at 44 years old
BC17-1	BBC	36	9 BC 1 GC 1 RC	RB IDC KB: DCIS	R B III	RB: T2 N1 Mo LB: T0 N0 M0	RB: 6N+/10N SG: 2N-/2N	RB: 25	+	+	NA	NA	Bilateral mastectomy/CT/RT/HT 28/01/1999 FAC Loco regional RT with a dose of 50Gy. Ovarian ablation by RT with a dose of 12Gy. HT tamoxifen 5 years	Treatment well-tolerated, 11 years of complete remission Esophageal Carcinoma at 48 years old. Disease progression, later cervical, bone, and liver metastases. Died at 50 years old
BC17-2	PABC UOQ (RB)	25	9 BC 1 GC 1 RC	IDC	II	T4bN1Mx	9N+/15N	55	60% +	80% +	–	20	Mastectomy (04/05/18) CT RT HT	CBC Bone metastases, Unplanned pregnancy during BC treatment Lung metastases at 27 years old
BC39	BC UOQ (LF)	29	No	IDC	III	T2 N0 M1	Not operated	Not operated	+	+	–	NA	4 lines of CT/HT CT: 1st line: 6 cycles FEC 75 +Zoledronic acid end November 2009 2nd line: 9 cycles Docetaxel until end 2011 3rd line Xeloda + zoledronic acid (3 cycles April August 2012) 4th line: Taxol-Bevacizumab-Zoledronic acid) then Zoledronic acid +Aromasine +Endoxan	Metastatic breast cancer at diagnosis (bone, liver) Disease progression Patient died at 35 years old after 4 lines of chemotherapy
BC95	BC JUQ (RB)	32	1 CRC	IDC	I	T1 N0 M0	9N–/9N	17	100% +	100% +	–	50%	Mastectomy, CT/HT 12/03/2012 6FEC100 then HT (Tamoxifen) and zoladex for 22 months	5 years after treatment, alive under regular surveillance
BC 225-1	BC UOQ LB	50	5 BC 2 EC 1 BrC	IDC	III	T2 N1 M0	1N+/13N	20	90% +	90% +	–	60%	Tumerectomy/CT/RT/HT 16/12/2016 FEC100 DOCETAXEl	Under regular surveillance

*BC, Breast Cancer; OC, Ovarian Cancer; GC, Gastric Cancer; RC, Renal Cancer; EC, Esophageal Carcinoma; BrC, Brain Cancer; ThC, Throat Cancer; IDC, Invasive Ductal Carcinoma; DCIS, diffuse carcinoma in situ; CT, chemotherapy; RT, Radiotherapy; HT, Hormonotherapy; RB, right breast; LB, left breast.; UOQ, upper outer quadrant; JUQ, junction of the upper quadrants; FAC, fluorouracil, doxorubicin, cyclophosphamide*.

Because of the rare genotype that we are investigating in this study, the number of cases reported in this study is too small to draw firm conclusions, however, there is evidences of an association between *Cis* DH and a relatively severe phenotype of the disease with early age of onset, high breast cancer grade and multiple cancer cases in the families.

In addition to the identified *Cis* DH pathogenic mutations, 21 SNPs have been detected on both *BRCA1* and *BRCA2* genes including 12 exonic and 9 intronic variants (Supplementary Table 1).

**Figure 3:**
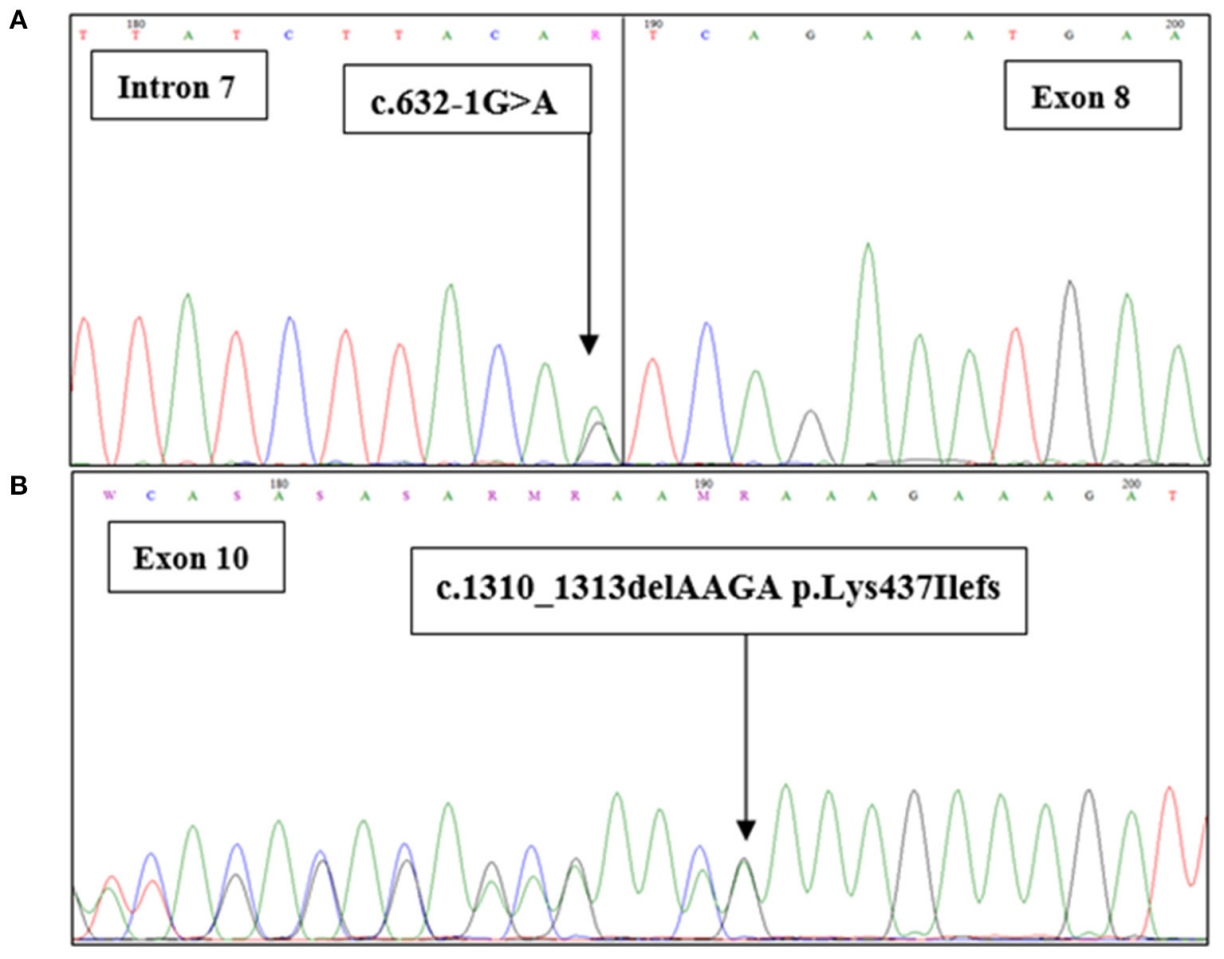
*BRCA2* sequence with the c.632-1G>A and c.1301-1304delAAAG mutations. Sanger chromatograms confirm **(A)** the presence of the c.632-1G>A mutation at the junction intron 7/exon 8 and **(B)** the c.1310_1313delAAGA (p.Lys437Ilefs) mutation in exon 10.

The co-occurrence of the two mutations on the same *BRCA2* allele raises questions about the functional significance of the splice site mutation (Figure 3). Three different *in silico* prediction tools have been used to assess the functional effect of this splicing mutation (Table 3). Results showed that c.632-1G>A seems to disturb the normal splicing by the loss of the WT acceptor site which may induce the skipping of *BRCA2*-exon 8.

**Table 3:** Summary of splicing predictions results.

	**Mutation taster**	**HSF**		**Alamut**
c.632-1G>A	Likely to disturb normal splicing (Acceptor lost)	Alteration of the WT acceptor site, most probably affecting splicing.		Most probably to induce skipping of exon 8
		1. HSF Matrices prediction variation (%)	2. MaxEnt prediction variation (%)	
		−32.22	−108.84	

Then we proceed with protein modeling as an additional functional analysis. Unfortunately, thec.632-1G>A splicing mutation does not appear in any regions or domains of the protein (described in uniport https://www.uniprot.org/uniprot/P51587). In addition, the existing 3D structures do not cover this region and therefore we were not able to generate a 3D model of the mutant with a good confidence rate.

For the RT PCR analysis, the cDNA of the *BRCA2* gene was amplified using several primer combinations flanking exons 5–9, 5–10, 6–10. Although electrophoresis gel showed a specific band for the resulting cDNA, sequencing analysis did not show any sequences supporting the skipping of the *BRCA2*-exon8 hypothesis.

### Haplotype Analysis

To investigate a possible founder effect, haplotype analysis was performed on 60 Tunisian breast cancer cases. In detail, 8 *BRCA2Cis* DH carriers, 3 *BRCA2* SH carriers, 4 relatives, 8 non-carriers, 24 breast cancer patients with unknown *BRCA* status and 13 healthy Tunisian women were investigated using the four selected STR markers. The D13S1246 marker was inconclusive and it was then excluded from the analysis. Table 4 summarizes the haplotype analysis for the *Cis* DH and SH carriers. Evaluation of the informative microsatellites was performed on probands and additional family members when possible. We identified a shared haplotype between all *Cis* DH and SH *BRCA2* mutation carriers (162-233-162) and absent or very rare in the remaining controls suggesting a founder effect of these two mutations.

**Table 4:** Haplotype analyses in the carriers of both *BRCA2* deleterious mutations.

**STR**		**D13S267**	**D13S171**	**D13S260**
**% HTZ**		68	71	77
**Range size**		148–162	227–241	158–173
BC6-1	*Cis* DH	150–**162**	**233**−243	**162**−170
BC6-4		162–**162**	**233**−247	162–**162**
BC6-5		162–**162**	**233**−247	**162**−162
BC17-1		150–**162**	233–**233**	**162**−166
BC39		**162**−164	**233**−235	**162**−172
BC95		162–**162**	233–**233**	162–**162**
BC225-1		150–**162**	**233**−243	158–**162**
BC225-2		**162–**162	231–**233**	**162**−172
BC245	SH	**162**−162	**233**−235	162–**162**
PEC009		150–**162**	229–**233**	**162**−170
PEC035		162–**162**	231–**233**	**162**−172

*In bold: (162-233-162) indicates the shared haplotype between all Cis DH and SH BRCA2 mutation Carriers. This haplotype is absent or very rare in the control group suggesting a founder effect of the two mutations in BRCA2*.

Moreover, all carriers of these two mutations are originating from the North Western and central regions of Tunisia. Of interest, *Cis* DH and SH carriers share the same haplotype suggesting that they are mostly inherited together.

## Discussion

Breast cancer is a malignancy with significant genotypic and phenotypic diversity (Almendro et al., 2014). It is now well-documented that a large proportion of North African breast cancer patients are relatively young (Corbex et al., 2014) with an average age at presentation of breast cancer patients a decade earlier than Caucasian individuals (Chouchane et al., 2013). Recent studies showed that 11% of Tunisian breast cancer patients are under 35 years old (Zehani et al., 2009). In addition, the majority of North African breast cancer patients are still diagnosed at late stages. Therefore, early disease onset and late diagnosis, both represent the main cause of high mortality among breast cancer patients in these populations. Given the fact that breast cancer in young women tends to behave more aggressively (Assi et al., 2013), an early diagnosis will result in reduced treatment, effective primary prevention and additional life years gained. Therefore, understanding the epidemiology-genetic factors behind the significant high number of young breast cancer patients in North African populations will promote early detection of the disease and will have a positive impact on the survival rates of affected patients.

In the present study, 6 Tunisian breast cancer patients harbor two pathogenic mutations on the same allele of *BRCA2* (c. 632-1G>A and c. 1310_1313 Del AAGA) have been detected using whole exome sequencing. These patients showed a significant early age of onset of the disease. Indeed, the mean age of onset was about 35.33 years in *Cis* DH, significantly younger than *BRCA* SH mutation carriers (48.1 years), *BRCA2* mutation carriers (47.5 years), and biallelic *BRCA* mutation carriers (44.6 years) (Rebbeck et al., 2016) suggesting an association of this genotype with an early age of onset.

Young age of onset was also observed in other unusual *BRCA* genotypes. Indeed, biallelic *BRCA2* mutations have been observed in a 31 years old female diagnosed with colorectal cancer (Degrolard-Courcet et al., 2014). Biallelic mutations in *BRCA1* have been reported in a female patient with epithelial ovarian carcinoma diagnosed at age 28 years (Domchek et al., 2013). Recent studies also showed that DH mutation carriers develop breast cancer at an earlier age and have more severe disease than SH cases (Lavie et al., 2011; Heidemann et al., 2012). Interestingly, Colombo et al. (2009) reported 4 *BRCA2 Cis* DH cases with a mean age of 34.

Therefore, we hypothesized that the development of early onset disease associated with co-occurrence of two deleterious mutations might be indicative of disruption of interactions between *BRCA2* and some key proteins resulting in an accumulation of DNA damages that makes the disease appear at an earlier age. In support of our hypothesis, Ashok Venkitaraman suggested that single heterozygosity in *BRCA* genes has a dosage effect sufficient to trigger low, but quantitatively significant, levels of genome instability. Alternatively, biallelic inactivation of *BRCA1* or *BRCA2* not only triggers profound chromosomal instability but also quickly leads to cell cycle arrest or apoptosis which results in an early presentation of the disease (Venkitaraman, 2019). In addition, *Cis DH* breast cancer patients reported in the current study, seem to present a high histological grade and a bigger tumor size with multiple breast cancer cases in the related family members suggesting an association between *Cis* DH and the phenotypic severity. A similar phenomenon, called negative complementation or metabolic interference, occurs when two alleles at the same locus interact to give a more severe phenotype in the compound heterozygote than in either homozygote (Johnson, 1980). For example, Abruptex (Abx) mutations of the Drosophila Notch gene fall into two genetic types, “enhancers” and “suppressors” of Notch. Homozygotes for either type are viable (characterized by gapping of the wing veins), yet compound enhancer/suppressor Abx heterozygotes are lethal (Kelley et al., 1987).

Of note, all reported patients with *Cis* DH come from a restricted geographic area suggesting a founder effect. We assume that there is no selection bias as patients from our cohort originated from different areas of Tunisia and were recruited from different oncological departments that have a national activity. Results from haplotype analysis confirmed this founder effect by the identification of a shared haplotype between all carriers of both *BRCA2* mutations, c. 1310_1313Del AAGA and c.632-1G>A.c.1310_1313DelAAGA is a known recurrent mutation in North African populations and has been already identified in Tunisia, Algeria and Morocco (Cherbal et al., 2012). Moreover, it was found in different European patients from Denmark, France, Belgium, Spain, and Italy and it has also been identified in a Korean patient (Díez et al., 2003; Thomassen et al., 2008; Caputo et al., 2012; Kim et al., 2012; Laarabi et al., 2017). This mutation was also found in several male breast cancer cases (Drusedau et al., 2013; Fourati et al., 2014; Silvestri et al., 2020). For the exon8- *BRCA2* c.632-1G>A mutation, it is described for the first time in Tunisia and in the North African region. It also appears to be rare in other populations since it was only reported in one patient with prostate cancer in the UK (Edwards et al., 2010). Different analysis showed that there is an over-representation of mutations in *BRCA2's* exons 7 and 8 in FA-D1 patients (Szabo et al., 2000; Rahman and Scott, 2007). Furthermore, despite the high frequency of *BRCA2* truncating exon 11 mutations in familial breast cancer there is currently no known FA-D1 patient with biallelic truncating mutations in exon 11 (Rahman and Scott, 2007). Interestingly, genotype-phenotype analyses of *BRCA2* breast cancer pedigrees mirror the observations in biallelic *BRCA2* mutation cases, with different cancer risks associated with monoallelic truncating mutations in exon 11 when compared with mutations located in 3′ or 5′ of this exon (Thompson et al., 2001; Lubinski et al., 2004).

In fact, genetic disease studies on North Africans are of keen interest because of their heterogeneous and admixed populations from African and European origins. The particular structure of the Tunisian population is also due to a relatively high rate of consanguinity that reaches 98.9% in some regions (Ben Halim et al., 2015). This clearly impacted the incidence rates of complex diseases as well as monogenic ones such as those predisposing to cancer (Romdhane et al., 2019). Studies on the impact of consanguinity on breast cancer incidences showed controversial results. Indeed, Gilani et al. showed that consanguinity is linked to a high overall risk of breast cancer (Masood Gilani and Kamal, 2004). However, other studies showed that a lower risk of breast cancer was reported in consanguineous families compared to non-consanguineous ones (Denic and Bener, 2001). Here we propose that consanguinity can also affect the early onset of breast cancer and other associated phenotypes. Indeed, a long history of consanguinity that may result in a genetic mixing that may cause the co-occurrence of more than one mutation on one allele such as the *Cis* DH observed in this study and that seems to be associated with increased likelihood of breast cancer incidence and early onset of the disease. In Ashkenazi Jewish, where a relatively high risk of consanguinity has been observed, double heterozygosity in both *BRCA1* and *BRCA2* mutations is frequently observed and usually, at least one of the DH mutations is a founder mutation. In this same context, Lavie et al. (2011) showed that a 1.85% prevalence of *BRCA1* and *BRCA2* DH in Ashkenazi Jewish patients with FA-D1 have a distinctively higher risk of specific cancers than patients in other Fanconi anemia complementation groups. Therefore, carrying a single mutant allele, biallelic or two monoallelic mutations are associated with a phenotypic variability, ranging from HBOC, fanconi anemia, to early age of breast cancer onset with multiple cancer cases in the family.

Interestingly, the identification of these phenotype-genotype correlations will help to provide appropriate and individualized genetic counseling, risk assessment and cancer surveillance not only for index cases but also to their relatives from both parental sides. Thus, carriers of DH mutations may benefit from an earlier start of surveillance, more intensive follow up care and/or an urgent prophylactic surgery. From a therapeutic point of view, while both *BRCA1* and *BRCA2* mutations sensitize cells to PARP inhibitors, the affected gene appears to have a modulating effect on sensitivity. Indeed, *BRCA1*-defective cells demonstrate a ~60-fold increase in sensitivity to Olaparib vs. *BRCA* wild-type cells, while the corresponding increase in sensitivity in *BRCA2*-defective cells is ~130-fold (Farmer et al., 2005). Moreover, loss of BRCA1 RING domain function appears insufficient to fully sensitize cells to PARP inhibition, while still predisposing to cancer development (Drost et al., 2011, 2016). Given the fact that the c.632-1G>A mutation identified in this study is located in the N-terminal region of BRCA2 protein corresponding to the RING domain of BRCA1, and because of the financial burden of targeted therapy use in routine clinical practice, identifying patients most likely to benefit from these drugs is of great importance.

Once confirmed in larger cohorts, our finding will also bring new insights to the scientific community that will either accept the *Cis* DH BRCA genotype that was largely debated, or it will help in reviewing the clinical actionability of the splicing c-632-1G>A *BRCA2* mutation that is still classified as pathogenic in ClinVar. Efforts made by *the Evidence-based Network for the Interpretation of Germline Mutant Alleles* (ENIGMA) in determining the clinical significance of sequence variants in *BRCA1, BRCA2*, and other known or suspected breast cancer genes represent an excellent initiative to overcome this challenge and will permit the reclassification of this mutation in order to avoid misdiagnosis and over treatment of breast cancer patients all over the world (Nielsen et al., 2018).

Furthermore, due to the high cost of the complete screening of the large *BRCA* genes, the screening of only recurrent or founder mutations using Sanger sequencing is a traditional genetic testing method in low and middle income countries (LMICs). This screening method is no longer concluding. Indeed, apart from the fact that this strategy may bias the frequency of *BRCA* mutations in a specific mutation, by doing so, we also risk to pass side of the double heterozygous or biallelic *BRCA* genotypes that seem to be associated with different phenotypic spectrum. This problem does not arise when using cost-efficient and sensitive new technologies such as gene panels or whole exome sequencing that extend the analysis to the whole *BRCA* and other breast cancer susceptibility genes regardless of previous identification of a specific mutation in the family.

Finally, as c.1310_1313DelAAGA is a founder mutation in North African populations, our results will have an impact not only on Tunisian cancer patients but on patients from other North African countries as well as cancer cases originating from other ethnic groups.

## Conclusion

Genetic studies of under-investigated populations may lead to the identification of unusual genotypes and phenotypes that provide novel insights and avenues for research regarding the complex biological functions of the *BRCA* genes. It also increases our understanding of the clinical basis of cancer predisposition which will help moving toward a more accurate and personalized clinical management of cancer patients.

## Data Availability Statement

All pathogenic mutations identified in this study with data related to these variants have been deposited in the public database “ClinVar” under the following link https://www.ncbi.nlm.nih.gov/clinvar/submitters/507986/. All remaining variations (non-pathogenic) identified within BRCA1 and BRCA2 genes have been provided in the Supplementary Material. Any additional details may be made available upon request after approval of our institutional review board.

## Ethics Statement

The studies involving human participants were reviewed and approved by Biomedical Ethics Committee of Institut Pasteur de Tunis (2017/16/E/hôpital a-m). The patients/participants provided their written informed consent to participate in this study. Written informed consent was obtained from participants for the publication of any potentially identifiable images or data included in this article.

## Author Contributions

YH prepared the study concept and design, supervised the study, did data analysis, data interpretation, drafted, and critically revised the manuscript. MB and NMi did the experiments, participated in participant recruitment, and participated in drafting and reviewing the manuscript. NMe, OJ, YH, MB, NMi, SBe, HBoua, JH, AZ, YB, HR, ND, HE, and SL contributed to data collection and clinical data analysis. SA, HBous, SBo, AH, and KR contributed to the results interpretation. SBo and SA contributed to the study concept, design and supervision. SA, SBo, and HBous critically revised the manuscript. All authors read and approved the manuscript.

## Conflict of Interest

The authors declare that the research was conducted in the absence of any commercial or financial relationships that could be construed as a potential conflict of interest.

## Publisher's Note

All claims expressed in this article are solely those of the authors and do not necessarily represent those of their affiliated organizations, or those of the publisher, the editors and the reviewers. Any product that may be evaluated in this article, or claim that may be made by its manufacturer, is not guaranteed or endorsed by the publisher.

## Abbreviations

*Cis* DH: *Cis* double heterozygosity
WES: Whole exome sequencing
NGS: Next Generation Sequencing
SH: single heterozygotes
TH: trans heterozygous
BC: Breast cancer
TNBC: Triple negative breast cancer
MBC: Male Breast Cancer
GC: Gastric Cancer
RC: Renal Cancer
EC: Esophageal Carcinoma
BrC: Brain Cancer
ThC: Throat Cancer
IDC: Invasive Ductal Carcinoma
DCIS: Diffuse carcinoma *in situ*
CT: Chemotherapy
RT: Radiotherapy
HT: Hormonotherapy
RB: Right breast
LB: Left breast
UOQ: Upper outer quadrant
JUQ: Junction of the upper quadrants.
