# Supplementary material for: Identification of BRCA2 Cis Double Heterozygous Breast Cancer Cases Using Whole Exome Sequencing: Phenotypic Expression and Impact on Personalized Oncology
Source: Front Genet. 2021 Aug 12;12:674990. doi: 10.3389/fgene.2021.674990 (PMC8397457; doi:10.3389/fgene.2021.674990)
Supplement: Supplementary file 1 [file Table_1.docx]

**Table S1. Variations identified in *BRCA1* and *BRCA2* genes**

| **Gene** | **Position** | **Variant ID** | **Genotype** | **Localisation** | **Nucleotide Change** | **Amino Acide Change** | **Frequency GnomAD** |
| --- | --- | --- | --- | --- | --- | --- | --- |
| ***BRCA1*** | 41277187 | rs799905 | HMZ | intronic | c.-20+101C>G | . | 0.000007794 |
|  | 41256090 | rs373413425 | HTZ | intronic | c.441+36_441+49delCTTTTCTTTTTTTT | - | 0.000005955 |
|  | 41256075 | rs72434991 | HMZ | intronic | c.441+63_441+64del | . | 0.003325 |
|  | 41245466 | rs1799949 | HTZ | exonic | c.2082C>T | S694S | 0.3486 |
|  | 41245237 | rs16940 | HTZ | exonic | c.2311T>C | L771L | 0.3422 |
|  | 41244936 | rs799917 | HMZ | exonic | c.2612C>T | P871L | 0.4127 |
|  | 41244435 | rs16941 | HTZ | exonic | c.3113A>G | E1038G | 0.3430 |
|  | 41244000 | rs16942 | HTZ | exonic | c.3548A>G | K1183R | 0.3486 |
|  | 41243190 | rs799916 | HMZ | intronic | c.4097-141A>C | . | 0.4490 |
|  | 41234470 | rs1060915 | HTZ | exonic | c.4308T>C | S1436S | 0.3433 |
|  | 41226601 | rs273900734 | HTZ | Intronic | c.4485-63C>G | - | 0.3164 |
|  | 41223094 | rs1799966 | HTZ | exonic | c.4837A>G | S1613G | 0.00007074 |
|  | 41219560 | rs8176235 | HTZ | intronic | c.5074+65G>A | - | 0.2493 |
|  | 41215825 | rs3092994 | HTZ | Intronic | c.5152+66G>A | - | 0.3139 |
| *BRCA2* | 32905265 | rs206073 | HMZ | intronic | c.793+98G>A | . | 0.9797 |
|  | 32906729 | rs144848 | HTZ | exonic | c.1114A>C | N372H | 0.2733 |
|  | 32912299 | rs543304 | HTZ | exonic | c.3807T>C | V1269V | 0.1754 |
|  | 32913055 | rs206075 | HMZ | exonic | c.4563A>G | L1521L | 0.000003989 |
|  | 32915005 | rs206076 | HMZ | exonic | c.6513G>C | V2171V | 0.00005166 |
|  | 32920844 | rs206080 | HMZ | intronic | c.6938-120T>C | . | 0.9799 |
|  | 32929387 | rs169547 | HMZ | exonic | c.7397T>C | V2466A | 0.9934 |

**Abbreviations: HTZ = heterozygous ; HMZ = Homozygous, NA : Non Available**
