# Supplementary material for: Identification of BRCA2 Cis Double Heterozygous Breast Cancer Cases Using Whole Exome Sequencing: Phenotypic Expression and Impact on Personalized Oncology
Source: Front Genet. 2021 Aug 12;12:674990. doi: 10.3389/fgene.2021.674990 (PMC8397457; doi:10.3389/fgene.2021.674990)
Supplement: Supplementary file 2 [file Image_1.pdf]

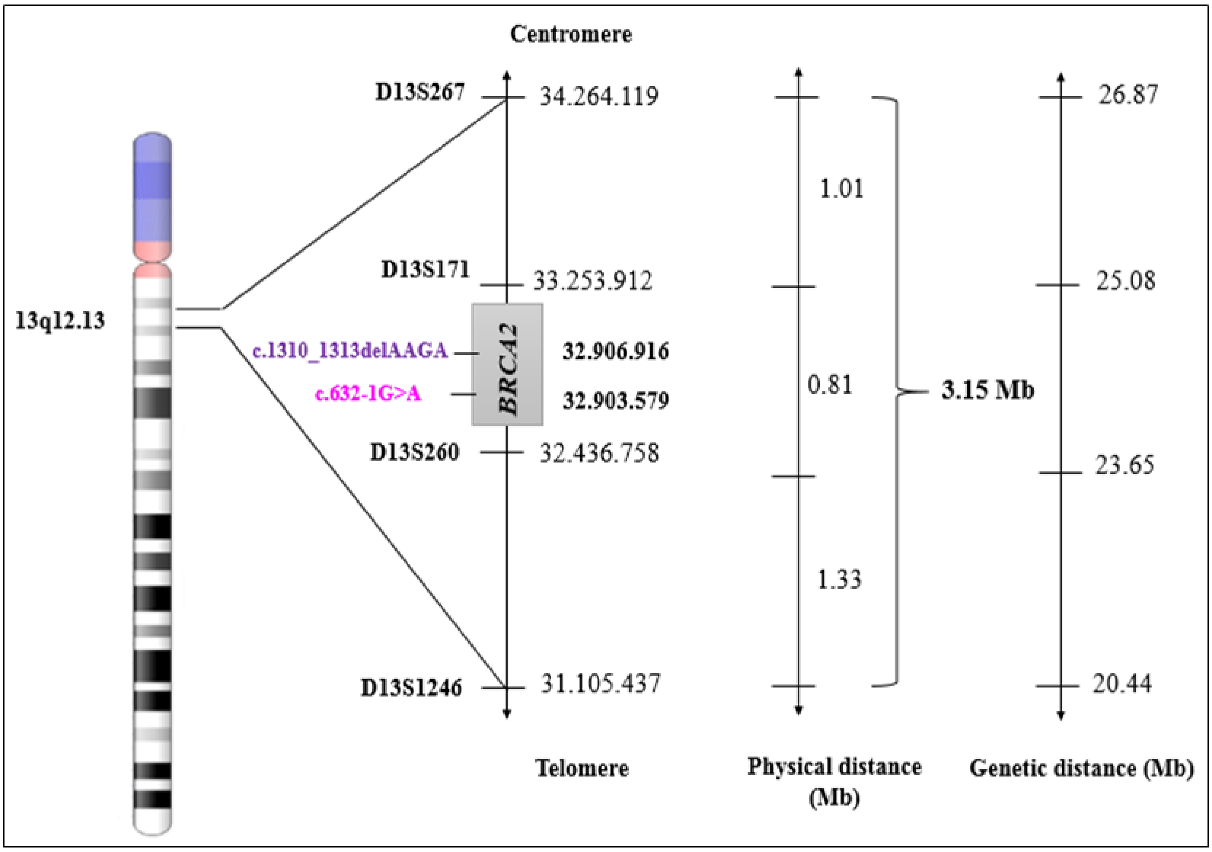

Supplementary Figure S1. Schematic representation showing the Short Tandem Repeats that were used for the haplotype analysis.
